# Influence of Gold Nanoparticle Incorporation Into Glass Ionomer Cement on Fibroblast Metabolic Activity, Surface Roughness and Hardness

**DOI:** 10.1155/ijod/3796785

**Published:** 2026-05-25

**Authors:** Areeba Zia, Andy Baldwin, Chibuzo Nlemorisa, Abdurahman Salem

**Affiliations:** ^1^ Queens Dental Sciences Centre, University of Greater Manchester (Formerly University of Bolton), Queen Street, BL4 7AH, Bolton, UK; ^2^ Centre for Clinical and Biomedical Sciences, University of Greater Manchester (Formerly University of Bolton), Queen Street, BL4 7AH, Bolton, UK

**Keywords:** cytotoxicity screening, dental restorative materials, fibroblast metabolic activity, glass ionomer cement, gold nanoparticles, root caries therapy, surface properties

## Abstract

**Objective:**

To assess the influence of gold nanoparticle (AuNP) liquid‐phase modification of glass ionomer cement (GIC) on fibroblast metabolic activity, alongside its effects on surface hardness and roughness, in an exploratory pilot in vitro study.

**Methods:**

GIC specimens were prepared using liquid mixtures containing 25% and 50% AuNP suspension within the liquid phase alongside an unmodified control. The exact AuNP concentration in the final set cement was not determined, and direct structural characterisation of nanoparticle incorporation was not performed. Cell viability was evaluated using the MTT assay at 24 and 72 h and data were analysed using the Kruskal–Wallis test followed by Dunn’s post hoc test. Surface hardness and surface roughness data were analysed using the Mann–Whitney *U* test. Significant difference was set at *p* < 0.05 for all tests. No formal a priori power calculation was performed.

**Results:**

At 24 h, the unmodified GIC group showed a mean cell viability of 3.86% ± 2.1%, while both experimental groups exhibited higher values: 15.32% ± 10.7% for the 25% AuNP–GIC group and 14.93% ± 12.6% for the 50% AuNP–GIC group. After 72 h, the 50% AuNP–GIC group maintained the highest cell viability: 8.63% ± 1.6%, which was significantly higher than the unmodified GIC group: 4.71% ± 2.6% (*p* < 0.05). However, all groups showed very low viability values overall, indicating marked cytotoxicity under the present experimental conditions. The AuNP‐containing groups therefore showed only a relative attenuation of toxicity compared with the unmodified GIC group. Incorporation of AuNPs did not significantly affect surface hardness (control: 37.16 ± 5.69 VHN; 50% AuNP–GIC: 35.42 ± 1.32 VHN) but resulted in a significantly smoother surface, with Ra values of 0.89 ± 0.40 µm for the control group and 0.48 ± 0.21 µm for the 50% AuNP–GIC group (*p* = 0.0104).

**Conclusion:**

Within the limitations of this exploratory pilot in vitro study, the 50% AuNP‐containing liquid formulation showed the most favourable overall findings, being associated with less severe reduction in fibroblast metabolic activity than the unmodified control, improved surface smoothness and no significant difference in hardness. However, these findings should be interpreted cautiously, and further biological and physicochemical characterisation studies are required before any conclusions can be drawn regarding compatibility or clinical applicability.

**Significance:**

In this preliminary in vitro study, AuNP‐containing liquid formulations used during GIC preparation were associated with less severe reduction in metabolic activity than unmodified GIC, together with reduced surface roughness. However, these findings should not be interpreted as evidence of acceptable cytocompatibility, and direct incorporation of nanoparticles into the final set cement was not confirmed. Further studies incorporating human oral cell models, complementary biological assays and physicochemical characterisation are needed before biological or clinical relevance can be established.

## 1. Introduction

Root caries are destructive lesions affecting the root surface and periodontium and represent a major cause of tooth loss, particularly in older adults. Among adults aged 60+, the median prevalence of root caries across studies was ~46% [1]. This trend is attributed to physiological changes in the root surface and increased periodontal exposure, which promote demineralisation [2]. Root surface lesions can be classified as carious or non‐carious; carious lesions arise from bacterial activity, whereas non‐carious ones result from mechanical or chemical processes such as abrasion, erosion or occlusal stress [3]. These conditions are commonly associated with gingival recession, the main predisposing factor for root caries [4].

The management of root caries begins with preventive strategies including oral hygiene reinforcement, dietary modification and topical fluoride application [5]. When restorative intervention becomes necessary, clinical challenges such as limited visibility, moisture control and access to subgingival margins make the selection of restorative materials critical [6]. The ideal material should demonstrate strong adhesion, biocompatibility and support for tissue regeneration [7].

Glass ionomer cement (GIC) is widely used for root caries restoration due to its fluoride release, chemical adhesion to dentine, and favourable biocompatibility [8–10]. Despite these advantages, the initial low pH of GIC during setting has been associated with transient cytotoxic effects and reduced fibroblast attachment. This raises concerns about early soft‐tissue responses around subgingival restorations [11–14]. To overcome these limitations, various additives such as silver, titanium oxide, zinc oxide and magnesium oxide have been incorporated into GIC to improve its mechanical and biological performance [15]. Salem et al. [9] found that adding type I collagen and Arg‐Gly‐Asp (RGD) peptide enhanced fibroblast bioactivity and vimentin expression without compromising strength. Similarly, nanohydroxyapatite (nHA) improved both bioactivity and compressive strength [16].

Recent advances in GIC modification have expanded beyond conventional inorganic additives to include plant‐derived bioactive agents, phytomedicine‐inspired modifiers and green‐synthesised nanoparticles. A recent comprehensive review highlighted growing interest in phytomedicine and green nanotechnology for enhancing the antimicrobial and selected physical/mechanical properties of GIC, while also emphasising the importance of careful formulation definition and direct material characterisation [17]. Similarly, a recent systematic review of propolis‐modified GIC reported promising antimicrobial effects but variable physico‐mechanical outcomes across formulations [18]. More recently, a Scientific Reports study investigated green‐synthesised TiO_2_ nanoparticle‐modified GIC and reported in vitro and in silico assessment of mechanical, physical and safety‐related properties [19]. Collectively, these studies demonstrate an increasing interest in multifunctional GIC formulations.

Gold nanoparticles (AuNPs) have gained attention for their excellent biocompatibility, low cytotoxicity and ability to promote cell proliferation and differentiation [20, 21]. Previous studies have shown that AuNPs enhance the growth and osteogenic differentiation of mesenchymal and adipose‐derived stem cells [22, 23]. Li et al. [24] reported that AuNPs significantly increased proliferation of human periodontal ligament stem cells and produced a modest enhancement of osteoblastic differentiation. Furthermore, the incorporation of AuNPs into dental biomaterials such as titanium implants, PMMA denture base resins and zirconia ceramics has been shown to enhance mechanical stability and/or biological performance, including hardness, dimensional stability, antimicrobial activity and osseointegration [25–29]. However, the incorporation of AuNPs into GIC remains insufficiently investigated. Therefore, this study aimed to evaluate the preliminary biological response and mechanical performance of AuNP‐modified GIC, with particular focus on L929 fibroblast metabolic activity, surface roughness and hardness. It was hypothesised that, under the present in vitro conditions, AuNP‐containing liquid‐phase modification of GIC would be associated with higher fibroblast metabolic activity compared with unmodified GIC.

## 2. Materials and Methods

### 2.1. Biological Testing

#### 2.1.1. Materials

ChemFil Superior GIC (Dentsply Sirona, Konstanz, Germany) was selected as the base material. Citrate‐stabilised AuNPs (20 nm; Sigma–Aldrich, USA) were dispersed in distilled water to prepare liquid mixtures containing 25% and 50% AuNPs (v/v). Distilled water alone (100%) was used as the liquid component for the control group. In the present study, the reported 25% and 50% AuNP values refer to the volume fraction of AuNP suspension within the liquid component used for mixing rather than the final mass concentration of nanoparticles in the set cement.

#### 2.1.2. Specimen Preparation

GIC powder and liquid components were mixed according to the manufacturer’s instructions. The control group was prepared using 100% distilled water as the liquid component. For the experimental groups, AuNP suspensions were incorporated into the GIC liquid by replacing an equivalent volume of distilled water. Specifically, the 25% and 50% AuNP groups were prepared by combining AuNP suspension with distilled water at ratios of 25:75 mL and 50:50 mL (v/v), respectively, while keeping the final liquid volume constant. Thus, the reported percentages represent the proportion of AuNP suspension within the liquid phase only, and the overall powder‐to‐liquid ratio was maintained across groups. These concentrations were selected as part of an initial exploratory (pilot) investigation to assess the feasibility of AuNP incorporation into GIC and its effect on biological response and surface properties. The exact mass concentration of AuNPs in the final set cement was not determined in the present study.

After thorough homogenisation, the mixtures were dispensed into PVC moulds (1 mm thickness × 7 mm diameter) placed between glass slides and allowed to set for 2 min.

Specimens were subsequently incubated in complete culture medium for 72 h at 37°C in a 5% CO_2_ environment to ensure acidity release and complete setting. They were then rinsed with phosphate‐buffered saline (PBS; Thermo Fisher Scientific, USA), air‐dried with sterile tissue paper and UV‐sterilised for 16 min (Lumily LED UV Sterilisation Unit) before being transferred to 48‐well plates (NuAire, USA) for biological evaluation.

#### 2.1.3. Cell Culture and Cytotoxicity Assay (MTT Test)

L929 mouse fibroblasts were cultured in DMEM with 10% FBS and 1% penicillin–streptomycin at 37°C in 5% CO_2_. Cells were seeded (2 × 10^4^ cells/500 µL) in 48‐well plates containing GIC specimens and incubated for 24 and 72 h. After each incubation period, media were removed and MTT solution was added for 3 h, followed by acidified isopropanol to dissolve formazan crystals. Absorbance was read at 590 nm (reference: 670 nm). Cell viability (%) was calculated using the following equation:

Cell viability %= ASample−AblankAcontrol Acell only−Ablank×100,

where *A*_sample_ represents wells containing cells exposed to the test specimen, *A*_blank_ represents reagent blank wells without cells and *A*_cell-only_ control represents wells containing cells cultured without any material specimen. In the present manuscript, the term control group in the Results and Tables refers to the unmodified GIC group, whereas the cell‐only control was used only for MTT normalisation.

L929 murine fibroblasts were used as a preliminary cytotoxicity screening model for in vitro screening assessment. Ten wells were used for each material group at each time point (24 and 72 h), and the reported values represent measurements from parallel replicate wells under the same experimental conditions.

#### 2.1.4. Surface Properties Testing

ChemFil Superior GIC (Dentsply Sirona, Germany) was used as the base material. AuNPs (20 nm, citrate‐stabilised; Sigma–Aldrich, USA) served as the modifying agent. The GIC powder and liquid were mixed according to the manufacturer’s instructions. For the control group, 100% distilled water was used as the liquid component. The 50% AuNP group was prepared by combining the AuNP suspension with distilled water in a 50:50 (v/v) ratio within the liquid phase. The 50% AuNP group was selected for surface integrity testing based on its higher MTT‐derived metabolic activity relative to the other tested groups in the cell viability assays. Although the powder‐to‐liquid ratio was kept constant, replacing part of the distilled water with AuNP suspension may have influenced the chemistry of the liquid phase and, consequently, the setting reaction and final material properties.

The mixture was homogenised and dispensed into PVC moulds (1 mm × 7 mm) positioned between glass slides to ensure uniform shaping. After initial setting, all specimens were stored in distilled water for 24 h. They were then polished uniformly to achieve a consistent surface quality prior to testing.

Due to the lack of access to scanning electron microscopy (SEM) and energy‐dispersive X‐ray spectroscopy (EDS) facilities within our laboratory, microstructural and elemental characterisation of the AuNP–GIC composite could not be performed in the present study. Consequently, nanoparticle incorporation, distribution and possible agglomeration within the GIC matrix were not directly verified.

#### 2.1.5. Surface Hardness

Vickers surface hardness was evaluated for both the control group (*n* = 5) and the 50% AuNP group (*n* = 5). No formal a priori power analysis was performed. The sample size was selected as part of this exploratory pilot study based on feasibility and consistency with previously published GIC microhardness studies, in which five specimens per group have commonly been used [30, 31]. Nevertheless, this relatively small sample size should be regarded as suitable for preliminary screening only, and the findings should be interpreted cautiously.

Hardness measurements were performed using a Duramin‐40 hardness tester (Struers A/S, Ballerup, Denmark) with a load of 100 g applied for 15 s. Three indentations were made at separate locations on each specimen, and the mean hardness value was calculated for statistical analysis. The Vickers microhardness (HV) was calculated using the following formula: HV = 1.8544 × *F*/*d* ^2^, where *d* is the mean diagonal length of the indentation and *F* is the applied force (*F* = *m* × *g*, with *g* = 9.81 N/kg).

#### 2.1.6. Surface Roughness

Surface roughness (Ra) was measured using a Profilm3D optical profilometer (Filmetrics, San Diego, CA, USA). Initially, 10 polished disc specimens were prepared for each group (*n* = 10). The sample size was selected as part of this exploratory pilot study based on feasibility and alignment with previously reported profilometric protocols for evaluating the surface roughness of GICs [32]. All specimens were visually inspected for surface integrity. Two specimens from each group were excluded due to the presence of surface defects, including air bubbles and cracks. Consequently, the final sample size for surface roughness analysis was eight specimens per group (control: *n* = 8; 50% AuNP: *n* = 8). Surface roughness measurements were obtained along a central trace across each specimen surface, and the mean Ra value for each specimen was recorded and used for statistical comparison. Given the final sample size (*n* = 8 per group), these data should also be interpreted as preliminary.

#### 2.1.7. Statistical Analysis

Statistical analyses were performed using IBM SPSS Statistics (IBM Corp., Chicago, IL, USA). Data normality was assessed using the Shapiro–Wilk test, supported by visual inspection of Q–Q plots for all outcome variables. The cell viability outcome was tested using the Kruskal–Wallis test, followed by Dunn–Bonferroni‐adjusted pairwise comparisons. Comparisons between 24 and 72 h within each material group were performed using the Mann–Whitney *U* test as measurements at the two time points were obtained from independent wells/specimens. Surface hardness and surface roughness data were analysed using the Mann–Whitney *U* test as comparisons were made between independent groups. Statistical significance was set at *p* < 0.05 for all analyses.

## 3. Results

### 3.1. Cell Viability

At 24 h, both experimental groups demonstrated higher cell viability than the control group, 3.86% ± 2.1%, with mean values of 15.32% ± 10.7% for the 25% AuNP‐GIC group and 14.93% ± 12.6% for the 50% AuNP‐GIC group. After 72 h, the 50% AuNP‐GIC group maintained the highest cell viability, 8.63% ± 1.6%, which was significantly greater than the control, 4.71% ± 2.6%, whereas the 25% AuNP–GIC group showed a reduction in viability, 3.68% ± 2.0% (Table 1).

**Table 1:** Mean and standard deviation (%) of cell viability at 24 and 72 h.

Group	Time (h)	Mean cell viability (% ± SD)
Control (unmodified GIC)	24	3.86 ± 2.1^a^
	72	4.71 ± 2.6^a^
25% AuNP‐GIC	24	15.32 ± 10.7^b^
	72	3.68 ± 2.0^a^
50% AuNP‐GIC	24	14.93 ± 12.6^ab^
	72	8.63 ± 1.6^b^

*Note:* Different superscript letters indicate statistically significant pairwise differences within the same time point based on Dunn–Bonferroni‐adjusted post hoc comparisons (*p* < 0.05).

At 24 h, a Kruskal–Wallis test demonstrated a statistically significant overall difference among groups (*H*(2) = 10.739, *p* = 0.0047). Dunn–Bonferroni‐adjusted pairwise comparisons showed a significant difference between the unmodified GIC group and the 25% AuNP–GIC group (*p* = 0.0047), whereas the comparisons between the unmodified GIC group and the 50% AuNP–GIC group (*p* = 0.0603) and between the 25% and 50% AuNP–GIC groups (*p* = 1.000) were not statistically significant. At 72 h, a Kruskal–Wallis test also demonstrated a statistically significant overall difference among groups (*H*(2) = 15.440, *p* = 0.00044). Dunn–Bonferroni‐adjusted pairwise comparisons showed that the 50% AuNP–GIC group had significantly greater cell viability than both the unmodified GIC group (*p* = 0.0123) and the 25% AuNP–GIC group (*p* = 0.00051), whereas no significant difference was observed between the unmodified GIC group and the 25% AuNP–GIC group (*p* = 1.000).

Comparisons between 24 and 72 h within each material group were performed using the Mann–Whitney *U* test. No significant difference was found between time points for the unmodified GIC group (*U* = 39.0, *p* = 0.436) or the 50% AuNP–GIC group (*U* = 62.0, *p* = 0.393). In contrast, the 25% AuNP–GIC group showed a statistically significant reduction in cell viability from 24 to 72 h (*U* = 97.0, *p* < 0.001) (Table 1).

### 3.2. Surface Properties Testing

The mean surface hardness of the control group was 37.16 ± 5.69 VHN, while the 50% AuNP‐GIC group showed a mean value of 35.42 ± 1.32 VHN. Surface hardness was compared between the control and 50% AuNP–GIC groups using the Mann–Whitney *U* test. No statistically significant difference was observed between the two groups (*p* = 0.6905) (Table 2).

**Table 2:** Mean surface hardness (VHN) values for control and 50% AuNP‐GIC groups.

Group	Mean ± SD (VHN)	Range (min–max)
Control	37.16 ± 05.69^a^	28.99–43.20
50% AuNP‐GIC	35.42 ± 01.32^a^	34.31–36.94

*Note:* Different superscript letters within the same column indicate statistically significant differences (*p* < 0.05).

The surface roughness decreased from 0.89 ± 0.40 µm in the control group to 0.48 ± 0.21 µm in the 50% AuNP‐GIC group. Surface roughness was compared between the control and 50% AuNP–GIC groups using the Mann–Whitney *U* test. A statistically significant difference was observed, with the 50% AuNP–GIC group showing significantly lower roughness values than the control group (*p* = 0.0104) (Table 3).

**Table 3:** Mean surface roughness (Ra) values for control and 50% AuNP‐GIC groups.

Group	Mean ± SD (µm)	Range (min–max)
Control	0.89 ± 0.40^a^	0.38–1.66
50% AuNP‐GIC	0.48 ± 0.21^b^	0.33–0.97

*Note:* Different superscript letters within the same column indicate statistically significant differences (*p* < 0.05).

## 4. Discussion

This exploratory pilot study found that the 50% AuNP‐containing liquid formulation was associated with higher MTT‐derived metabolic activity than unmodified GIC, together with lower surface roughness and no significant difference in measured surface hardness. These findings should be interpreted cautiously in view of the marked cytotoxicity observed across all groups, the limited biological assessment, and the absence of direct material characterisation.

The relatively high standard deviations observed in the 24 h AuNP‐modified groups indicate increased variability in early cellular responses. This variability may be attributed to heterogeneous cell‐nanoparticle interactions during the initial exposure period, differences in nanoparticle dispersion at the material surface, and intrinsic cell‐to‐cell variability in nanoparticle uptake. A previous study by Aberg et al. [33] has demonstrated that nanoparticle internalisation can vary substantially between individual cells, even within the same population, leading to heterogeneous early biological responses. This interpretation remains speculative and requires further investigation.

The observed differences between the tested liquid formulations suggest that formulation‐dependent effects may have occurred under the present experimental conditions [34, 35] However, because nanoparticle incorporation into the set cement was not confirmed, these differences should not be interpreted as evidence of a verified dose‐dependent AuNP effect. Instead, they may reflect changes in the liquid‐phase composition and its influence on the setting reaction or material behaviour. The overall decline in viability from 24 to 72 h may be attributed to ion release and pH alterations associated with glass ionomer maturation in static culture conditions [36, 37].

The higher MTT‐derived metabolic activity observed in the 50% AuNP–GIC group, relative to the unmodified GIC group, should be interpreted cautiously and within the limitations of this preliminary screening model. Since the biological evaluation was restricted to L929 fibroblasts and a single MTT assay, no conclusions can be drawn regarding proliferation, cellular attachment, extracellular matrix interactions, signalling pathways, or other underlying biological mechanisms. These were not investigated in the present study.

Despite the overall decline in viability over time, the 50% AuNP–GIC group consistently showed higher metabolic activity than the unmodified GIC group in relative terms within this preliminary screening model. Accordingly, the study hypothesis is supported only in comparative terms and not as evidence of acceptable cytocompatibility or broader biological compatibility.

Regarding surface properties, the reduction in roughness observed in AuNP‐modified GIC may reflect changes within the material matrix under the present experimental conditions [38–40]. However, because direct material characterisation was not performed, the smoother surface cannot be attributed confidently to confirmed nanoparticle incorporation, interstitial void filling, or improved nanoparticle–matrix interfacial compatibility, and may instead reflect changes in liquid‐phase chemistry during preparation. Although the reduction in surface roughness observed in the AuNP‐modified GIC was statistically significant, its clinical significance should be interpreted cautiously. Surface roughness values above ~0.2 µm have been associated with increased bacterial adhesion and plaque accumulation. In this study, AuNP incorporation reduced roughness from 0.89 ± 0.40 µm in the control group to 0.48 ± 0.21 µm in the 50% AuNP‐containing formulation group, representing a smoother surface under the present experimental conditions. Although both values remain above the plaque‐retention threshold, the smoother surface observed in the 50% AuNP‐containing formulation group may still be considered a favourable material finding under the present experimental conditions; however, its clinical relevance remains uncertain [41, 42].

In this study, the incorporation of AuNPs did not significantly influence the HV of the material. Both control and 50% AuNP–GIC groups yielded values consistent with those previously reported for modern GIC systems, which typically range from ~35–60 VHN depending on the formulation [43]. The absence of a significant difference suggests that AuNP incorporation did not measurably reduce hardness under the present experimental conditions and may indicate that the modification did not markedly disrupt the acid–base setting reaction or polysalt matrix formation responsible for the mechanical behaviour of GIC [44, 45]. Although the hardness values are at the lower end of the reported range for GICs, they remain within the reported ranges for these materials [12]. However, this finding alone is insufficient to infer long‐term clinical performance for the present formulation.

Overall, these findings suggest that modifying the GIC liquid phase with AuNPs improved relative metabolic activity and surface properties compared with the unmodified material. Further studies using human oral cell models, complementary biological assays, and physicochemical characterisation are needed to clarify the clinical relevance and underlying mechanisms of these findings.

### 4.1. Limitations

This study is limited by the use of a single biological model and a single assay. L929 murine fibroblasts were used as a preliminary bioactivity screening model, and the MTT assay reflects cellular metabolic activity only rather than direct attachment, adhesion or morphology. Accordingly, the present findings should be regarded as preliminary and hypothesis‐generating rather than confirmatory. The study was also exploratory in nature, and no formal a priori power analysis was performed. For the MTT assay, 10 wells were used per material group at each time point, and the results represent replicate well measurements within the same experimental setup rather than repeated independent biological experiments. In addition, the exact mass concentration of AuNPs in the final set cement was not determined, and SEM/EDS characterisation was not performed; therefore, nanoparticle incorporation, distribution, and possible agglomeration within the GIC matrix were not directly verified. For this reason, the observed effects cannot be attributed conclusively to confirmed AuNP incorporation into the set material. Additional biological assays, larger studies, human gingival fibroblast cell models, and physicochemical characterisation would strengthen future evaluation of AuNP‐modified GIC.

## 5. Conclusion

Within the limitations of this exploratory pilot in vitro study, modification of GIC with AuNP‐containing liquid formulations was associated with less severe reduction in metabolic activity compared with unmodified GIC and improved surface smoothness without reducing measured surface hardness under the present test conditions. The 50% AuNP–GIC formulation showed the most favourable overall findings among the tested groups. However, further biological and physicochemical studies are required before definitive conclusions can be drawn regarding clinical relevance or the precise mechanistic role of AuNP incorporation.

## Author Contributions

Areeba Zia, Chibuzo Nlemorisa, Andy Baldwin and Abdurahman Salem: investigation, methodology, formal analysis, visualisation, writing – original draft, writing – review and editing. Abdurahman Salem: conceptualisation, project supervision.

## Funding

This study was not funded by any agency in the public, commercial or nonprofit sectors.

## Disclosure

All authors have read and approved the final manuscript.

## Ethics Statement

This study was approved by the Research Ethics Committee at the University of Bolton (now University of Greater Manchester) (Certificate Number AUTONOHP00587). All methods were performed following the declaration and the ethical guidelines of the University of Bolton Research Ethics Committee. No human participants or tissue were involved in the study.

## Conflicts of Interest

The authors declare no conflicts of interest.

## Data Availability

The datasets generated and analysed during the current study are available from the corresponding author upon reasonable request.
